# The Irony of Iron – Biogenic Iron Oxides as an Iron Source to the Ocean

**DOI:** 10.3389/fmicb.2015.01502

**Published:** 2016-01-06

**Authors:** David Emerson

**Affiliations:** Bigelow Laboratory for Ocean SciencesEast Boothbay, ME, USA

**Keywords:** iron-oxidation, iron-oxidizing bacteria, iron cycle, biogenic iron, hydrothermal vents, hydrothermal diffuse flow, sediment transport, marine iron limitation

## Abstract

Primary productivity in at least a third of the sunlit open ocean is thought to be iron-limited. Primary sources of dissolved iron (dFe) to the ocean are hydrothermal venting, flux from the sediments along continental margins, and airborne dust. This article provides a general review of sources of hydrothermal and sedimentary iron to the ocean, and speculates upon the role that iron-cycling microbes play in controlling iron dynamics from these sources. Special attention is paid to iron-oxidizing bacteria (FeOB) that live by oxidizing iron and producing biogenic iron oxides as waste products. The presence and ubiquity of FeOB both at hydrothermal systems and in sediments is only beginning to be appreciated. The biogenic oxides they produce have unique properties that could contribute significantly to the dynamics of dFe in the ocean. Changes in the physical and chemical characteristics of the ocean due to climate change and ocean acidification will undoubtedly impact the microbial iron cycle. A better understanding of the contemporary role of microbes in the iron cycle will help in predicting how these changes could ultimately influence marine primary productivity.

## Introduction

An oceanographer on the deck of a research vessel in the center of a mid-ocean gyre adds a milliliter of a dilute solution of iron chloride to a tank of seawater, she has collected and creates an algae bloom. Three thousand meters below the ship, an undersea hydrothermal vent emits iron-rich vent fluids feeding a unique community of lithoautotrophic iron-oxidizing bacteria (FeOB) that grow in a microbial mat a meter or more thick. Yet, due to the chemistry of iron and the physical structure of the ocean, virtually none of this iron reaches the sea surface; such is the irony of iron.

The Earth’s core is composed of iron and it is the fourth most abundant element in the crust; yet the primary production of phytoplankton in 30–40% of the world’s oceans is limited by iron. In the presence of oxygen at oceanic pH, reduced, dissolved ferrous iron [Fe(II)] will oxidize spontaneously to ferric Fe(III) iron, a one electron transfer, and Fe(III) will instantaneously react with water to form an iron oxyhydroxide with a vanishingly small solubility. In the photic zone of the well-oxygenated modern ocean these oxides precipitate to the depths leaving the surface waters anemic. Interestingly, the reason we refer to anemia at all is because nature has taken advantage of iron’s facility for interacting with oxygen to make it central to heme that carries oxygen in our blood. Plants, including single celled algae, take advantage of the redox properties of iron to aid multiple steps in the photosynthetic conversion of light energy to chemical energy (Raven et al., 1999); therefore, a lack of available iron limits their growth. The reaction center of nitrogenase, the enzyme responsible for nitrogen fixation, also requires iron (Anderson et al., 2013); hence, biological N_2_-fixation can become iron-limited as well.

The dynamics of the iron cycle in the ocean are thus of great importance. In the coastal ocean, terrestrial inputs from rivers and turbulent mixing of sediment layers provide an adequate source of iron, such that coastal surface waters are rarely iron-limited. In the open ocean, well clear of the continental shelves, Aeolian dust blowing off the continents is the primary source of iron to the surface waters (Moore and Braucher, 2008). This is why the mid-Atlantic, downwind of the Sahara, has some of the highest surface iron concentrations (∼0.76 nM) of any ocean (Raiswell and Canfield, 2011). The converse is true throughout the higher latitudes of the southern ocean, where geography and climate limit the input of dust-borne iron (Martin et al., 1990), and surface ocean iron concentrations may be 5–10 times below North Atlantic values. At these concentrations iron can become the limiting nutrient for the growth of phytoplankton. Mineral debris transported via icebergs that calve from the Antarctic continent can partially supplant Aeolian dust as an iron source to the southern ocean (Raiswell and Canfield, 2011). Nonetheless, from a global perspective, these are among the most anemic waters in the world with correspondingly low rates of primary productivity.

Remarkably, specific groups of bacteria and archaea learned long ago to take advantage of the one electron transfer between Fe(II) and Fe(III) to capture energy to support their growth (Emerson et al., 2010). The most prolific of these FeOB require Fe(II) concentrations in the micromolar range, orders of magnitude higher than are in typical seawater. They grow best at low oxygen concentrations where there is less competition with chemical iron oxidation (Emerson et al., 2010). This restricts FeOB to areas of the ocean floor where such conditions prevail around areas of hydrothermal activity, or sediments that promote the iron cycle.

## Hydrothermal Vent Associated Iron-Utilizing Communities

In the deep ocean, hydrothermal vents are generally recognized as the most significant source of iron, and are the most well-studied habitats for FeOB. Vent systems make up only a tiny percentage of the areal extent of the seafloor yet emit remarkable quantities of iron and sulfur minerals with current estimates for iron-release being on the order of 50 Gg yr^-1^ (Raiswell and Canfield, 2011). A relatively recent discovery is that these iron-rich plumes can be transported 1000s of kilometers from their source. This can be due to nano- and micro-particulate iron sulfide minerals derived from high temperature black smokers (Yücel et al., 2011; Gartman et al., 2014), or fine particulates of iron bound to organic matter (Toner et al., 2009; Bennett et al., 2011; Wu et al., 2011). In either case, these nanoparticles contribute to the pool of dissolved iron (dFe) in the ocean. dFe is commonly defined as iron that passes through a 0.2 μm filter; irregardless of its oxidation state or elemental composition (Raiswell and Canfield, 2011).

These findings controvert the conventional wisdom that vent-sourced iron quickly precipitates from the water column, and has led to a re-evaluation of the potential for hydrothermal iron sources to contribute to the overall iron budget of the ocean (Tagliabue et al., 2010; Fitzsimmons et al., 2014). Furthermore, it has also been assumed that the chemocline that creates a strong physical separation of surface and deep waters would minimize input of hydrothermally derived iron to the surface ocean, except in areas of strong upwelling. Modeling evidence from the southern ocean suggests that a relatively small, but still significant fraction of this deeply sourced iron could end up in the photic zone, and act as an ‘iron buffer’ to dust borne iron deposition (Tagliabue, 2014; Tagliabue et al., 2014). The guiding hypothesis presented by Tagliabue et al. (2014) is that hydrothermal sources are a more constant dFe source over geological time scales, than dust borne deposition that is strongly influenced by periods of glaciation and deglaciation with concomitant changes in sea level.

Given the importance of hydrothermal vents as a dFe source for the global ocean, it is reasonable to ask how microbes involved in the iron cycle, and FeOB, in particular, may exert influence on this process. These bacteria grow most prolifically at colder (<100°C) diffuse flow sources, as opposed to the high temperature (≥350°C) black smokers. These diffuse vents are often more highly enriched in Fe(II) than reduced sulfur species (Emerson and Moyer, 2010). At these sites, microbial mats composed largely of biogenic iron oxides, referred to here as iron mats, can be centimeters or more thick. These iron mats harbor unique microbial communities dominated by the Zetaproteobacteria, a class of Proteobacteria that is found preferentially in high iron habitats in the ocean, and whose cultured members are lithotrophic FeOB (Scott et al., 2015). Members of this group produce diverse, filamentous micrometer-scale biogenic iron oxides, such as helical stalks and hollow, tubular sheaths that are easily recognized by light microscopy (Chan et al., 2010). These structures prevent the cells from becoming entombed in an impermeable iron-oxide shell. In addition, because the microbes are attached, the filaments provide a means for cells to maintain position in optimal fluxes of Fe(II) and O_2_ required for growth.

Detailed mineralogical analysis of these biogenic oxides find the predominant mineral is an amorphous ferrihydrite that is uniquely stable, and slow to undergo diagenesis to more crystalline iron oxides (Toner et al., 2008; Toner, 2012). It is not clear whether this stability is due to silicification, organic components produced by the bacteria, or some combination thereof. At present, our understanding of the organic components of biogenic iron oxides is limited to spectroscopic studies that indicate they are most likely biopolymers of acidic polysaccharides (Chan et al., 2010) that can also adsorb and assimilate organic carbon from their surroundings (Bennett et al., 2014). A more detailed biochemical analysis of the composition of stalks, sheaths, or other filamentous structures produced by marine FeOB has yet to be attempted.

Genomic analysis of Fe-oxidizing Zetaproteobacteria reveals subunits of genes annotated as cellulose synthases are quite common in gene involved in exopolymer production (Emerson, unpublished results). While cellulose polymers have yet to be found in cultured, stalk-forming Zetaproteobacteria, one function of these types of exopolymers could be to aid in buoyancy of the cells. Consistent with this, an analysis of intact microbial iron mats from Loihi Seamount revealed a remarkable level of coordination in the growth of stalk-producing FeOB to produce cohesive cauliflower-like structures that grow away from rock surfaces and toward the water column (Chan et al., submitted). This mechanism of growth is advantageous in keeping the cells at the mat front close to oxygenated seawater, while still being exposed to Fe(II) coming from vent fluid. The important point is that this growth strategy results in relatively buoyant, biogenic iron oxides that have the potential to become entrained in the water column.

The fate of these biologically precipitated iron oxides is poorly understood. When produced in the vicinity of black smokers, it is possible they can become entrained in the higher temperature plumes. One study that looked at particle sedimentation rate from vent plumes that arose from black smokers at 9°N on the East Pacific Rise found evidence of Zetaproteobacteria in a trap further ‘down-plume,’ but not in a trap closer to the vents (Sylvan et al., 2012). This is consistent with the relatively buoyant nature of biogenic oxides, and indicates their potential to be transported in the water column. In general, however, there is little evidence for the presence of known FeOB in plumes associated with black smokers (Dick et al., 2013). This is in keeping with the finding that the majority of vent-associated iron mats either occur in systems not associated with black smokers, or are distal enough from the smokers themselves that entrainment of biogenic oxides in plumes is limited.

At hydrothermal vents where microbial iron mats are actively accreting, presumably there is continual shedding of biogenic oxides from mat into the surrounding seawater. Detailed ultra-structural analysis of the iron oxides produced by stalk-forming and sheath-forming FeOB reveal the oxides are composed of nanometer scale fibrils that wind together to produce larger, micron scale structures (Chan et al., 2009, 2010). These are often referred to as hydrous ferric oxides (Fortin and Langley, 2005; Suzuki et al., 2011). They have large reactive surface areas, but are also delicate, with the potential to breakdown to nanoparticulate oxides of a size consistent with the dFe in the ocean (Ferris et al., 2015). The extent of this process, or how much these biogenic oxides could contribute to dFe budgets is unknown; nor is it known if biogenic oxides are a good iron source for phytoplankton and other microbes.

Our understanding of the flux and dynamics of biogenic iron oxide production is also rudimentary, as are estimates of actual *in situ* activities of FeOB. Glazer and Rouxel (2009) used an *in situ* microsensor to conduct a high resolution depth profile of an iron mat at Loihi Seamount that revealed steep gradients of Fe(II) within a 2–3 cm thick microbial iron mat, and a net transport of Fe(II) out of the mat. They found that as O_2_ concentration of the overlaying seawater decreased, there is a greater flux of Fe(II) out of the mat. Based on modeling of O_2_ and Fe(II) fluxes in Loihi iron mats it was estimated that a range of 1 × 10^7^–4 × 10^8^ cells cm^-2^ yr^-1^ could be supported on Fe(II)-fueled growth (Glazer and Rouxel, 2009). As yet, there are no experimentally based estimates of microbial iron oxidation rates from deep-sea hydrothermal iron mats. Cell-specific iron oxide production rates will be important for understanding the overall potential for biogenic iron oxides to contribute to the pool of dFe in the deep ocean.

## The Importance of Diffuse Hydrothermal Flows as a Source of Biogenic Iron

Most *a priori* assumptions are that black smoker systems are the major source of iron to the ocean; however, recent assessments indicate diffuse hydrothermal flow systems may contribute as much or more dFe to the ocean. Compared to black smokers, it is difficult to constrain the total flux of hydrothermal fluids from diffuse systems, since they can vary widely in flow rates and temperature. Studies from the Juan de Fuca Ridge hydrothermal vent system estimated ≥90% of the mass flow was sourced from diffuse flows (Rona and Trivett, 1992; Baker et al., 1993; Schultz and Elderfield, 1997). Remarkably, a recent modeling analysis by German et al. (2015) suggested that up to 90% of the dFe that is supplied by hydrothermal venting to the distal ocean originates from these diffuse sources. A recent analysis of dFe concentrations from a transect of the southern Pacific ocean stretching 4,000 km west from the East Pacific Rise ridge axis, revealed significantly higher dFe concentrations in deep ocean water than would have been predicted based on standard models of iron deposition from hydrothermal systems (Resing et al., 2015). The authors attribute much of this dFe to diffuse flow sources. These results confirm the probability that dFe, often stabilized by ill-defined organic ligands, can be transported over basin-scale distances in the ocean. Furthermore, these authors estimate the global supply of dFe from ridge axes may be three to 4 Gmol per year, nearly fourfold higher than previous estimates.

Another challenging facet of diffuse venting systems that harbor microbial iron mats is that they can be challenging to find, especially if they are not associated with high temperature vent fields. These systems can exhibit small temperature anomalies and produce minimal buoyant plumes that are often used to detect hydrothermal vents. A particularly striking case was the fortuitous discovery of meter thick microbial mats of iron and manganese-oxidizing bacteria associated with the base of Loihi Seamount at a depth of 5,000 m (Edwards et al., 2011). This ecosystem covered 100s of m^2^, and had localized temperature anomalies ranging from a few tenths to a few degrees C above the background seawater. The extent of diffuse and ultra-diffuse sources that are geographically isolated from high temperature systems (e.g., >100°C) along tectonically active ridge crests or around seamounts is an unknown. Is it possible there are numerous, low temperature diffuse, iron-rich ‘springs’ dotted along the ocean ridges and around active seamounts, and, if so, how important a role might they play in the biogeochemical cycle of iron at the seafloor? When coupled with the capacity for FeOB to produce hydrous, nanoparticulate iron oxides, this opens the possibility for biogenic oxides to make a significant contribution to dFe in the global ocean.

## Sedimentary Iron Sources that Fuel the Microbial Iron Cycle

Sedimentary sources of iron are an important source of dFe to the ocean, especially along continental margins. Recent modeling efforts suggest that the amount of dFe (∼109 Gmol yr^-1^) contributed by continental shelves to the global ocean is several times greater than previous estimates (Dale et al., 2015). Some marine sediments contain substantial amounts of reactive iron oxides that may support levels of iron reduction that rival or exceed rates of sulfate reduction (Thamdrup, 2000). Although sulfate is the major anion in anoxic marine environments that microorganisms use as an electron acceptor for anaerobic respiration, Fe-reduction is the thermodynamically favored process and so can outcompete sulfate reduction if reactive iron oxides are sufficiently available (Lovley and Phillips, 1987). A confounding factor for this biological process is that the hydrogen sulfide that results from sulfate reduction can chemically react with poorly crystalline iron oxides resulting in the formation of iron sulfides (Thamdrup and Canfield, 1996). Furthermore the reactivity of different oxidation states of iron with organic matter as well as soluble Fe(III) can further complicate biotic versus abiotic iron chemistry (Beckler et al., 2015). Nonetheless, it is assumed that microbes play an important role in iron reduction, although few marine iron-reducing bacteria have actually been isolated. As a result, our knowledge about their physiology and ecology, compared to terrestrial strains, is lacking. For example, little is known about the metabolic flexibility of marine iron reducers. Presumably a significant percentage of them can reduce sulfate as well as iron oxides, but are there appreciable numbers of obligate marine iron reducers in marine sediments?

A number of measurements have been made for the benthic flux of iron from continental margin sediments from the west coast of the United States, with other measurements along the Peru Margin and South Africa, as well as sediments in the embayments of Arctic glaciers (Severmann et al., 2010; Noffke et al., 2012; Homoky et al., 2013; Wehrmann et al., 2014). To cite specific examples, measured porewater concentrations of Fe(II) ranging from around 5 to 180 μM have led to modeled iron eﬄuxes that can range from ∼2.5 μmol m^-2^ day^-1^ up to 100 μmol m^-2^ day^-1^ (Homoky et al., 2012). Even higher fluxes of iron have been reported from sediments associated with an oxygen minimum zone off the coast of Peru, where porewater concentrations of Fe(II) up to 90 μM were measured (Dang et al., 2011; Noffke et al., 2012). An important point is that these higher porewater Fe(II) values are well within the known minimal concentration (1–10 μM) of Fe(II) that would support the growth of FeOB, so long as there is a sufficient Fe(II) flux. The highest porewater Fe(II) concentrations tend to be within a couple centimeters of the sediment surface, and implies relatively steep gradients of Fe(II) could provide an energy source for FeOB growing in the micro-oxic zone near the sediment surface. The slope or trajectory of these gradients in the sediments will be governed by the O_2_ demand related to the availability of organic matter, as well as the O_2_ concentration of the overlying water.

The most striking evidence for iron oxidation is observation of rust-colored oxides, for example the thick floculant mats that form around hydrothermal vents. If the relative rates of iron reduction are high, either through microbial reduction or chemical reduction with sulfide, then iron oxides will be reduced quickly and there will be minimal accumulation of iron oxides. Furthermore, currents and wave action will tend to disperse hydrous ferric oxides that accumulate on surface sediments. These processes could lead to a cryptic iron cycle. A key question then revolves around the extent to which populations of FeOB are present, and active. Even if the numbers of FeOB in these sediments are relatively low, e.g., 10^5^–10^6^ cells cc^-1^ compared to the total cell populations of 10^8^–10^9^ cells cc^-1^, they could be oxidizing a significant amount of Fe(II). Ultimately, their activity will determine the balance of the iron cycle between reduction and oxidation, and constrain the release of either Fe(II) or hydrous ferric oxides from the sediments.

While the role of FeOB in these processes is still largely unknown, there is a growing body of evidence indicating that they are present and active. Studies of bio-corrosion of mild steel in both Maine and China have shown that FeOB can rapidly colonize steel surfaces (Dang et al., 2011; Mcbeth et al., 2011). These results indicate there must be a local reservoir of FeOB, most likely in shallow marine sediments, or nearby brackish environments (Mcbeth et al., 2013), or through aquifer-based intertidal mixing zones. In support of this latter source, a recent study of an intertidal zone in Delaware found significant levels of biological iron oxidation, and evidence for marine FeOB along a beach front where a freshwater aquifer rich in Fe(II) was upwelling into the ocean (McAllister et al., 2015). A recent modeling study of submarine groundwater discharge along continental margins estimated the flux of these groundwaters could be three to four times greater than riverine inputs into the ocean (Kwon et al., 2014). It is well known in the water industry that aquifers often contain appreciate levels of iron, and FeOB are common (Emerson and de Vet, 2015). Thus it is possible these anoxic or hypoxic waters could be a significant source of Fe(II) as well as FeOB and iron-reducing bacteria, that could feed an active microbial iron cycle in near shore sediments.

A survey of the Levantine margin off the coast of Israel found visual evidence for iron oxide deposits on the seafloor at depths of 300–800 m (Rubin-Blum et al., 2014). Molecular analysis revealed the presence of putative FeOB belonging to the Zetaproteobacteria as dominant members of these communities. The iron-oxidizing communities were associated with animal burrows, where presumably bioturbation and ventilation of the sediments led to a situation allowing for transport of O_2_ into anoxic sediments enriched in Fe(II) providing an ideal habitat with counter-gradients free Fe(II) and O_2_. Worm burrows in shallow sediments can also be a source of iron oxide precipitates where FeOB are present (McAllister et al., 2015). These initial studies suggest biological Fe-oxidation in marine sediments is far more common than previously realized. If this phenomenon is widespread, then FeOB may play a significant role in transforming iron, and controlling both the kinetics and the quality of iron transported into the water column.

## The Microbial Iron Cycle in a Changing Ocean

Continuing build-up of carbon dioxide in the atmosphere is a primary driver of climate change, global warming, ocean acidification, and sea-level rise. The impact of these affects on hydrothermally derived iron to the global ocean will come about as a result of changes in oceanic circulation patterns that are difficult to predict, but could have major impacts, either positive or negative, on delivery of iron to the ocean’s anemic regions. A better understanding of biogenically produced iron associated with hydrothermal vents will aid our understanding of how this could ultimately affect iron distribution patterns. In terms of near shore benthic habitats, climate change will have more direct impacts, through increased hypoxia and acidification. In general, anoxic and hypoxic conditions will favor iron reduction and the release of Fe(II) into the water column. Hypoxia will favor microbial Fe-oxidation. Likewise, the kinetics of Fe-oxidation are most sensitive to pH, thus decreasing pH will enhance the stability of Fe(II) making it more available for microbial Fe-oxidation. Thus one might predict a strengthening of the iron cycle, which would have important feedbacks to the supply of dFe to open, sunlit ocean, where primary productivity would be enhanced. On the other hand, hypoxic bottom waters will lead to die-offs of sedimentary in-fauna leading to decreased bioturbation and bioirrigation of sediments. Could this more than counter-balance the factors of hypoxia and lower pH? Finally, the iron and phosphorus cycles are linked due to the capacity for iron-oxyhydroxides to strongly adsorb phosphate. Although, it is known that iron minerals associated with hydrothermal plumes play a role in scavenging phosphorus (Dick et al., 2013), specific interactions between biogenic iron oxides and phosphorous have not been studied in marine systems. Recent work in terrestrial environments has shown the presence of biogenic iron oxides can significantly influence phosphorus dynamics (Baken et al., 2015) thus it is reasonable to expect similar interactions could be important in the ocean. These and many other questions about the roles of microbes in the marine iron cycle are worthy of study, and will undoubtedly reveal even more ironies about iron.

## Conflict of Interest Statement

The author declares that the research was conducted in the absence of any commercial or financial relationships that could be construed as a potential conflict of interest.
